# Emotional Intelligence and Anxiety in Nursing Students in Special Services Clinical Practices

**DOI:** 10.3390/jintelligence14060099

**Published:** 2026-06-04

**Authors:** María Anunciación Jiménez-Marcos, Ana María Insausti-Serrano, Ana Beatriz Bays-Moneo, Natalia Domínguez-Sanz, Izaskun Montori-Rodrigo

**Affiliations:** Faculty of Health Sciences, Public University of Navarra, Avda Barañáin, s/n, 31008 Pamplona, Navarra, Spain; ana.insausti@unavarra.es (A.M.I.-S.); anabeatriz.bays@unavarra.es (A.B.B.-M.); natalia.dominguez@unavarra.es (N.D.-S.); izaskun.montori@unavarra.es (I.M.-R.)

**Keywords:** emotional intelligence, anxiety, nursing, students, clinical practicum

## Abstract

Nursing students in their training process often suffer from anxiety due to stressful situations, and emotional intelligence can help them to manage these situations. The aim of this study is to analyse the associations between the dimensions of perceived emotional intelligence and anxiety in students undergoing their training cycles in different special services in order to check if there are differences between them. It is an observational, cross-sectional and correlational study with a sample of 85 nursing students who had not received training in emotional intelligence. Two measurement instruments were used: the Trait-State Anxiety Inventory (STAI) to assess anxiety and the Trait Meta-Mood Scale (TMMS-24) to measure EI. Data were analysed using Pearson’s coefficient when the distribution was normal, and Spearman’s coefficient in the non-normal distribution. The results showed in the group—ER-Emergency and Oncology—there was a significant negative relationship between state and trait anxiety and emotional understanding and regulation. In contrast, in the Primary Care setting there was also a positive relationship between emotional perception and trait anxiety. The study concludes that nursing students who understand and manage their emotions may have a lower risk of anxiety. Furthermore, if they identify emotions appropriately, the risk of suffering from anxiety in the long term may be lower. This finding was observed when the student did the internship in Primary Care. So there is a difference depending on the clinical context.

## 1. Introduction

Most university students begin their Bachelor’s degree at the age of 18, in the transition between adolescence and young adulthood. This is a life stage involving many changes, representing the beginning of independence, moving from the smaller family environment into larger society (Moshman, 2005).

They face multiple challenges, such as adapting to the preparatory role of schools and universities, a critical development period (Worsley et al., 2021), where great changes take place, which might bring about stressful situations. How they manage them will affect their life in one way or another.

Nursing students face other related challenges in the clinical context, such as dealing with violence, fear and anxiety, social-cultural challenges and environmental tensions, among others (Jahrami et al., 2023). Furthermore, they are increasingly concerned about their professional future (Nelson et al., 2023).

These challenges can contribute to nursing students experiencing significant levels of anxiety. Consistent with this, scientific evidence indicates that this group presents high anxiety levels during their training process (Thi Nhi et al., 2022).Therefore, mental health concerns can be raised in the nursing student collective, as this not only affects their personal well-being but also their professional performance (Karaca et al., 2019).

In healthcare work, interpersonal relations are important for professionals to perform their duties. Part of a nurse’s work is emotional; therefore, when nurses are aware of their emotions and manage them appropriately, this helps to reduce their stress and anxiety levels (Hong & Lee, 2016), and consequently they experience greater well-being and are able to provide higher-quality care to their patients (Carvalho et al., 2018).

That is why future nursing professionals need to know how to manage situations that can affect them emotionally. This ability to manage emotions is known as emotional intelligence (EI).

When it comes to conceptualising EI, there are various models that explain the construct, including the following:

- Salovey and Mayer (1990) define it as the ability to manage feelings and emotions, to distinguish between them, and to use this knowledge to guide one’s own thoughts and actions, dividing it into three areas or branches.
- According to Goleman (1996), it is the ability to recognise our own feelings and those of others, to motivate ourselves, and to manage our emotions both personally and in our interpersonal relationships.
- Bar-On (2006) defines it as a set of non-cognitive competencies, skills and abilities that influence success in dealing with context-related problems.

According to Boyatzis et al. (2000), EI is structured around key components: self-awareness, self-management, social awareness, social management and empathy. Developing these components requires an understanding of how situations affect them emotionally, the origin of those emotions, and how they can be managed (Salovey et al., 1995).

Among nursing students, scientific evidence has shown that those who are able to manage their emotions effectively experience positive effects on their health (Alconero-Camarero et al., 2018; Lee & Gu, 2013), alleviating the negative effects of stress (Alconero-Camarero et al., 2018). Furthermore, a review conducted by (Lewis et al., 2017) revealed that educational intervention can improve nursing students’ physical and psychological well-being during clinical placements.

Studies related to EI and anxiety in the nursing student collective have focused on academic and/or care contexts, although the clinical scenario of the practicum was not specified (Lewis et al., 2017). Consequently, this study examines the possible associations between the dimensions of perceived emotional intelligence (PEI) and anxiety among fourth-year nursing students undertaking clinical placements in various clinical departments, intending to identify differences that will enable a deeper understanding of the role of emotional intelligence in anxiety.

## 2. Materials and Methods

### 2.1. Study Design

This is a transverse, observational and correlational study, which measured anxiety levels and the PEI of nursing students while they took part in the clinical practicum cycle.

### 2.2. Participation and Data Collection

As the group of students undertaking placements in specialist services consists of fourth-year students undertaking a Bachelor’s degree in nursing, the session introducing the practical placement was used to explain the aim of the study and its relevance to them. Out of a total population of 220 students from two consecutive academic years, 110 initially agreed to take part in the study, of whom 85 completed it.

With regard to the sample size for each group, it should be noted that, in the fourth year of the Bachelor’s degree in nursing, approximately 16 students per cohort undertake clinical placements in the Intensive Care Unit. For this reason, it was decided to collect data over two consecutive academic years, which amounts to a maximum of approximately 30 students. Similarly, around 32 students per year undertake placements in the Oncology department; therefore, even if data collection was extended to two academic years, the maximum achievable sample size would be 64 participants. The same situation applies to the Accident and Emergency and Primary Care departments. Consequently, these structural limitations justify the final sample size.

The data were collected at the end of the practicum cycle, considering that the students had amassed enough experience in the practicum context by this point. This way the conclusions will provide a more accurate reflection of the current situation in the 4 clinical contexts.

The inclusion criteria: 4th-year students, doing practicum within the specialised hospital departments of “Accident and Emergency”, “Intensive Care Unit (ICU)”, “Oncology” and “Primary Care (PC)” who had not received EI training within their nursing degree education.

### 2.3. Measuring Instruments

Two instruments were used in this study: the State-Trait Anxiety Inventory (STAI) scales and the Trait Meta-Mood Scale (TMMS-24).

At the time of measuring, the students anonymously answered the STAI, which was developed by Spielberger (1972) and adapted and validated in Spanish by the TEA Ediciones studies section in 1988 (Spielberger et al., 2015). The STAI is the most widely used questionnaire for assessing anxiety levels; it is a simple, brief and useful self-report tool for evaluating anxiety levels. It comprises two scales that measure trait anxiety, a person’s tendency to feel anxious and state anxiety, which refer to how the person feels at the time of the assessment.

The instrument demonstrates robust psychometric properties in a sample of Spanish university students. It shows high internal consistency, with Cronbach’s alpha values of 0.8 for state anxiety and 0.88 for trait anxiety (Fonseca-Pedrero et al., 2012). These values indicate high reliability in measuring the construct.

Each of the STAI scales comprises 20 items, each one with Likert-type scale response options ranging from 0 to 3 points.

This assessment instrument was used to ascertain the different likely responses to psychological stress with different levels of state anxiety and identify the percentage of participants who might be at risk of suffering from anxiety disorders.

The PEI was also measured quantitatively during the time spent in the clinical practicum. The TMMS-24 assessment instrument was used, developed by Salovey et al. (1995), and modified and adapted into Spanish by Fernández-Berrocal et al. (2004), which has demonstrated good psychometric properties for use in the Spanish university population, with internal consistency estimates for the subscales all above 0.85. Furthermore, in a Spanish-speaking nursing student population, Cronbach’s alpha coefficients of 0.88, 0.89 and 0.86 were obtained for the respective dimensions (Espinoza et al., 2015), indicating adequate internal consistency and high reliability.

This instrument comprises 24 items where the subjects assess how much they agree on a Likert-type scale with five options (1 = strongly agree; 5 = strongly disagree). The questionnaire is divided into three sections: emotional perception, which implies the capability to deal with feelings; emotional comprehension, referring to a good understanding of their emotions; and emotional regulation, which refers to the capability to handle emotional states.

The PEI dimensions are categorised in three levels: perception of the emotion (“0” must improve; “1” suitable; “2” must improve due to too much attention), understanding of the emotion (“0” must improve; “1” suitable; “2” excellent) and their regulation (“0” must improve; “1” suitable; “2” excellent).

### 2.4. Data Analysis

First, a general analysis was performed on overall contexts, subsequently divided into clinical contexts. To check whether there was a relationship between the PEI and anxiety levels in the clinical practicum, a correlation analysis was performed because the dimensions of these variables were quantitative, and depending on the distribution of their values, the Pearson coefficient was used when the distribution was normal and the Spearman coefficient was used in a non-normal distribution.

As the aim of the study was not to establish causal relationships but to explore associations between variables, no attempt was made in this context to control for confounding variables using adjusted models.

All analyses used the IBM SPSS Statistics for Windows programme, Version 27.0. Armonk, NY, USA. The results were considered significant for *p* < 0.05.

### 2.5. Ethical Considerations

Participation was informed and voluntary, requiring written consent.

The study was conducted in accordance with the Declaration of Helsinki and approved by the Ethics, Animal Experimentation and Biosecurity Committee at the Public University of Navarre (code PI-025/16).

## 3. Results

The study sample will now be described. The participants, 85 fourth-year nursing students, carried out their clinical placements in four clinical contexts, distributed as follows: 27 in Oncology, 26 in Primary Care, 21 in the Accident and Emergency department and 11 in Intensive Care Units.

The overall mean age was 22.87 ± 4.925 years. Subdivided by setting, the average age was 24.85 ± 7.325 years in Primary Care; 24.09 ± 6.549 years in the Intensive Care Unit; 21.57 ± 1.363 years in the Accident and Emergency Department; and in 21.48 ± 0.975 years Oncology. In terms of gender distribution, the majority were women (*n* = 79).

With regard to the descriptive statistics for the STAI and TMMS-24 questionnaires, the mean and standard deviation were analysed where the data followed a normal distribution. In cases where the data did not follow a normal distribution, percentiles were used as a descriptive measure.

As can be seen in Table 1 and Table 2, on the one hand, the variables with a normal distribution showed values within the mean. On the other hand, for those variables that did not follow a normal distribution, more than half of the participants obtained scores considered to be within the normal range.

With regard to the variables used to measure anxiety and emotional intelligence, before the practicum finished, it was observed that, across the whole group, both the state anxiety and the trait anxiety present a significant moderate and negative relationship with comprehension (−0.431; −0.569) and regulation (−0.583; −0.557). However, a similar relationship is not found with emotional attention (Table 3).

Regarding the relationships between the dimensions of each variable analysed in this section, the following results were obtained.

In the PEI, comprehension and emotional regulation demonstrate a moderate and significantly positive dependent relationship (0.506) (Table 3).

In addition, it is important to highlight that the state anxiety and trait anxiety variables demonstrate a highly dependent relationship (0.787) (see Table 3).

Throughout the study, students who felt high levels of state anxiety might also feel high levels of trait anxiety or vice versa.

The results obtained in the four clinical contexts being researched are presented and analysed below.

In the context of the Accident and Emergency department, it was shown that students who experienced the highest levels of state anxiety and trait anxiety were less able to understand and manage their emotions as shown by the significant moderate–high negative dependent relationships that state anxiety and trait anxiety have with comprehension ((−0.503); (−0.604)) and with emotional regulation ((−0.597); (−0.466)) (Table 4).

On the other hand, the comprehension and regulation dimensions of PEI present a significant moderate and positive relationship. The high levels of trait anxiety are associated with lower comprehension and emotional management in the case of state anxiety and trait anxiety as shown by their significant strong and positive dependent relationship (Table 4).

In the field of Oncology, a moderately negative relationship is observed between state anxiety and comprehension (−0.541) plus a strongly negative relationship between state anxiety and emotional regulation (−0.611) (Table 5).

In the PEI dimension, there is a significant moderate and positive correlation between comprehension and emotional regulation (Table 5).

In addition, a significant moderate negative relationship is identified between trait anxiety and emotional regulation (Table 5).

On the other hand, a moderate negative relationship is established between state anxiety and comprehension (−0.541), and a strong and negative relationship is found between state anxiety and emotional regulation (−0.611) (Table 5).

Regarding the PEI dimensions, a significant moderate positive correlation is found between comprehension and emotional regulation (Table 5).

The results obtained in the PC context demonstrate that the students who managed to understand their emotions also managed to deal with them appropriately (0.551) (Table 6).

It was also shown that students with high levels of anxiety paid more attention to their emotions, although they found it hard to understand and handle them appropriately. Furthermore, it was observed that students who did not understand the reason behind their emotions properly could not manage these emotions.

It is also seen that students who experienced the highest levels of state anxiety had difficulties understanding and managing their emotions.

Additionally, it is highlighted in the context of PC, in the Accident and Emergency group and in the overall data set, that participants with the highest levels of trait anxiety also presented high levels of state anxiety, and vice versa.

Finally, no significant results were observed in the ICU group.

## 4. Discussion

The discussion begins with an analysis of the results obtained from the sample as a whole, before delving deeper into the examination of these results, taking into account the different specific contexts that have been studied. The available scientific evidence shows that emotional intelligence in nursing students plays an important role in managing and coping with anxiety. In this regard, several studies have indicated that adequate levels of EI are associated with better emotional regulation and, therefore, lower levels of anxiety. An example of this is the study conducted by Zhang et al. (2016), which analysed nursing students from all years of the degree programme and observed a negative and statistically significant correlation between EI and psychological anxiety. These findings suggest that poor emotion management can promote the development and intensification of psychological anxiety in this group, reinforcing the importance of promoting emotional skills during university nursing education.

When comparing the results of this study with previous research that has used the TMMS-24 assessment tool, a consistent trend can be observed that reinforces the relationship between adequate emotional understanding and regulation and lower levels of anxiety. Specifically, studies such as that by Irarrázabal et al. (2020) showed that students with greater skills in identifying, understanding, and regulating their emotions had significantly lower levels of anxiety. Similarly, Mesa Catro (2019) confirmed these same findings in nursing professionals, highlighting that EI also acts as a protective factor against anxiety in the workplace. These results are consistent with those obtained in the current study, which adds strength to the existing evidence. However, a relevant difference with respect to the studies cited is that, in the present research, this relationship manifested itself specifically during the clinical internship period, a particularly emotionally demanding context, which underscores the importance of emotional regulation in real healthcare situations.

Likewise, this study has observed a positive relationship between the different dimensions of perceived emotional intelligence, highlighting in particular the association between the dimension of emotional understanding and emotional regulation. This finding suggests that students who show a greater ability to identify and understand their emotional states and those of others also tend to have a greater ability to regulate those emotions appropriately. Similar results have been described in previous studies (Aradilla-Herrero et al., 2012; Augusto-Landa et al., 2009), in which this relationship has been validated in samples of nursing students, providing consistency and empirical support for the findings of the present study. Taken together, these results seem to indicate that a higher level of emotional understanding translates into a greater ability to manage emotions adaptively, an aspect that is particularly relevant in training and care contexts characterised by a high emotional load.

Correspondingly, when analysing the results corresponding to the different dimensions of anxiety, it can be observed that students with higher levels of state anxiety also tend to show higher levels of trait anxiety. This finding suggests that experiences of situational or momentary anxiety may be consistently reflected in an individual’s overall perception of anxiety, indicating the possible stability of these levels over time. Consequently, although in the short term students may experience episodes of anxiety related to specific situations, in the long term they may develop a tendency to maintain anxiety levels above those considered normal, which could harm both their emotional well-being and their academic and professional performance. This pattern reinforces the importance of implementing emotional management strategies and prevention programmes that allow for early intervention, promoting the development of emotional regulation skills that reduce the likelihood of anxiety becoming chronic.

In order to understand more precisely the relationship between anxiety and perceived EI, the group of students was analysed separately, which allowed an individualised assessment of the behaviour of each subgroup and the observation of possible contextual differences.

Relationships were observed in Accident and Emergency, Oncology and Primary Care. A negative relationship was seen between state anxiety and comprehension, and with emotional regulation, and the same occurs with trait anxiety and comprehension, and with emotional regulation.

These findings are particularly relevant when considering that nursing professionals working in Accident and Emergency departments are exposed to high levels of stress, likely caused by the heavy workload of unexpected, critical, and urgent situations they must attend to (Jiao et al., 2023). If even experienced professionals in this field experience stress and anxiety, it is understandable that students doing their placements in these same areas also require specific attention in terms of developing their emotional skills. Supporting this idea, a previous study conducted with emergency nursing students showed that those with difficulties understanding and managing their emotions lacked professional fulfilment, one of the key dimensions of burnout syndrome (Nespereira & Vázquez, 2017). This finding suggests that acquiring and strengthening emotional understanding and regulation skills can not only help to reduce anxiety in students but also prevent negative consequences associated with stress and professional well-being in their future clinical practice.

Another study focusing on nursing students doing their placements in Oncology showed that those students who shared their experiences of complex and emotionally difficult situations with clinical staff, such as the rapid deterioration of paediatric patients’ health, were able to manage their emotions more effectively (Mirlashari et al., 2017). This finding highlights the importance of social support and professional guidance as strategies that facilitate emotional regulation in highly emotionally charged contexts. Sharing difficult experiences not only allows students to process and understand their own emotional reactions but also contributes to the development of coping and resilience skills, which are essential for their professional and personal development.

The studies in both Accident and Emergency and Oncology mention that considering their emotions helps the student to improve their emotional well-being. This study is different due to the clear relationship appreciated between comprehension and regulation of emotions and anxiety, both in Accident and Emergency, and Oncology. This means that, during practicum, state anxiety and trait anxiety tend to drop when students understand and manage their emotions suitably.

On the other hand, in PC, there is also a clear relationship between comprehension and regulation of emotions and anxiety, although this differs from previous studies because there is also a relationship, in this case, a positive one, between anxiety and emotional perception. This suggests that when the students tend to experience high levels of anxiety, they may focus excessively on their own emotions, which will harm their ability to regulate their emotions.

Consequently, in this study, the PC context is seen to be the most influential in terms of students managing their emotions.

The study by Yebra Delgado et al. (2020) has similar outcomes to the results of this study. In this case, the relationship was between the three dimensions of burnout syndrome and the three dimensions of PEI. Nevertheless, in the PC collective, where students are seen to pay too much attention to their emotions, the risk of suffering from BS increases. This finding is consistent with the findings of the present study, although in this case it refers specifically to anxiety levels among nursing students. The fact that these results are observed in this context and not in others may be because in Oncology and Accident and Emergency departments, situations are more complex and demanding. This could encourage students to develop coping strategies, helping them to put their emotions into perspective. In contrast, in PC, where situations are not usually as critical, students with a greater tendency towards anxiety have fewer opportunities to face this type of experience and, therefore, to develop such coping strategies.

It should also be added that in all these scenarios (Accident and Emergency, Oncology and PC), a strong positive relationship appears between state anxiety and trait anxiety.

If the student tends to perceive anxiety regularly in their everyday life, then they might end up perceiving it in the long term. This perception will get in the way of how they understand and manage their emotions.

In respect of the relationships between the dimensions of the PEI, Oncology and PC are the contexts in which a greater understanding is found to lead to greater emotional regulation and lower anxiety. These results may be due to the fact that these are contexts in which the student is potentially closer to the patient and can empathise more with them and with the people around them. This could facilitate introspection regarding how these situations affect them. Being able to comprehend these situations leads them to try to understand what they are feeling. These are departments in which nurses witness situations that cause them to reflect deeply on their own lives.

Finally, although the ICU is an emotionally charged environment, the results obtained were not statistically significant. This finding may be due to the small sample size; consequently, it should be interpreted with caution. Future research should consider including a larger number of participants to reflect on the reality of the phenomenon under analysis more accurately.

In line with the findings of this research, authors such as Li and Hasson (2020) recommend paying greater attention to the emotional well-being of nursing students’ emotional states, and this current study has shown that managing emotions reduces anxiety levels for nursing students and so EI should be included in the nursing degree programme.

## 5. Conclusions

In conclusion, the differences observed depend on the clinical context, with PC showing the greatest discrepancies compared to the others. In particular, students with a tendency towards anxiety tend to focus excessively on their emotions, which will have a negative impact on their learning process. Furthermore, a nursing student who can identify their emotions, understand them and manage them appropriately will also run a lower risk of suffering from anxiety.

The limitations of this study include the small sample size and the predominance of female participants, which reflects both the limited available population and the characteristic demographic composition of the group studied. However, its potential lies in qualitative analysis because the information collected by the instruments enables further in-depth study on individual strategies adopted in the clinical contexts being studied.

## 6. Proposal for EI Training in the Primary Care Practicum

Based on the findings of this study, Primary Care is identified as an ideal setting for the development of emotional intelligence. For this reason, a proposal is put forward for a future recommendation in this context during the clinical placement cycle, as part of the nursing degree programme at the Public University of Navarra (UPNA). 

The practicum learning process involves three key figures: the clinical supervisor, the academic tutor (coordinator and facilitator of the Practice Integration Seminars (SIPA)), and the students themselves.

The following section outlines the **proposed methodology for the integration seminars (SIPA) and the EI**: 

The SIPAs act as spaces for continuous monitoring and evaluation. Eight group sessions are proposed, which integrate the EI as follows:

**Session 1**: The entire internship process will be explained, beginning with a presentation of the assigned internship services, the learning outcomes to be achieved upon completion of the internship at the Health Centre, the reflective journal to be kept by writing down experiences each week so that they can be analysed in each session, the evaluation process, and clarification of any doubts or questions that may arise.

**Intermediate sessions (6 sessions)**: The first session in this block focuses on exploring the constructs of EI in Salovey et al.’s (1995) model and their direct impact on student health and the development of technical and cross-cutting skills. To encourage participation, the study begins with reflective journals, providing ongoing feedback and taking care to avoid causing negative feelings (Naber & Markley, 2017). In order to guarantee anonymity and confidentiality, the journals are written in capital letters, allowing each student to analyse a classmate’s account impartially. This anonymous group analysis provides a safe space where students can express their experiences more freely.

In the following sessions, the intervention continues with progressive training in emotional self-awareness through reading diaries and identifying the triggers of each emotion related to healthcare practice.

**Final session**: The closing seminar will focus on applied emotional regulation, providing tools to address and resolve critical scenarios that compromise the emotional balance of future nurses.

## Figures and Tables

**Table 1:** Descriptive statistics for STAI dimensions.

Dimensions STAI	N	Mean (SD)	P50 (Median) (P25/P75)
SA (total group)	85	-	14 (7/22.5)
TA (total group)	85	17.1 (7.4)	-
SA (A&E group)	21	20.6 (12)	-
TA (A&E group)	21	21.9 (9.7)	-
SA (Oncology group)	27	14.6 (10)	-
TA (Oncology group)	27	20.3 (9.6)	-
SA (PC group)	26	-	13 (5/19)
TA (PC group)	26	15.8 (4.4)	-

Note: SA (state anxiety), TA (trait anxiety), N (sample number), SD (standard deviation), P (percentile).

**Table 2:** Descriptive statistics for TMMS-24 dimensions.

Dimensions TMMS-24	N	Mean (SD)	P50 (Median) (P25/P75)
A (total group)	85	-	28 (22.5/32,)
C (total group)	85	-	29 (26/32)
R (total group)	85	-	27 (24.5/31)
A (A&E group)	21	26.9(6.3)	-
C (A&E group)	21	-	29(24/39)
R (A&E group)	21	25.6(3.9)	-
A (Oncology group)	27	-	29 (25/33)
C (Oncology group)	27	28.7 (5.6)	-
R (Oncology group)	27	-	28 (26/31)
A (PC group)	26	25.1(6.5)	-
C (PC group)	26	29.1(4.3)	-
R (PC group)	26	28.9(5.4)	-

Note: A (attention), C (comprehension), R (regulation), N (sample number), SD (standard deviation), P (percentile).

**Table 3:** Correlations between PEI dimensions (TMMS-24) and anxiety levels (STAI State and Trait) total group.

Overall Group	Attention	Comprehension	Regulation	State Anxiety	Trait Anxiety
Attention	-				
Comprehension	0.237 *	-			
Regulation	0.000	0.506 **	-		
State Anxiety	0.112	−0.431 **	−0.583 **	-	
Trait Anxiety	0.200	−0.569 **	−0.557 **	0.787 **	-

*Note:* ** *p* < 0.01; * *p* < 0.05.

**Table 4:** Correlations between PEI dimensions (TMMS-24) and anxiety levels (STAI State and Trait) in the A & E context.

Accident & Emergency	Attention	Comprehension	Regulation	State Anxiety	Trait Anxiety
Attention	-				
Comprehension	0.064	-			
Regulation	−0.103	0.429	-		
State Anxiety	0.272	−0.503 *	−0.597 **	-	
Trait Anxiety	0.125	−0.604 **	−0.466 *	0.837 **	-

*Note:* ** *p* < 0.01; * *p* < 0.05.

**Table 5:** Correlations between PEI dimensions (TMMS-24) and anxiety levels (STAI State and Trait) in the Oncology context.

Oncology	Attention	Comprehension	Regulation	State Anxiety	Trait Anxiety
Attention	-				
Comprehension	0.323	-			
Regulation	−0.053	0.429 *	-		
State Anxiety	−0.079	−0.541 **	−0.611 **	-	
Trait Anxiety	0.125	−0.702 **	−0.588 **	0.708 **	-

*Note:* ** *p* < 0.01; * *p* < 0.05.

**Table 6:** Correlations between PEI dimensions (TMMS-24) and anxiety levels (STAI State and Trait) PC context.

PC	Attention	Comprehension	Regulation	State Anxiety	Trait Anxiety
Attention	-				
Comprehension	0.097	-			
Regulation	−0.207	0.551 **	-		
State Anxiety	0.240	−0.556 **	−0.517 **	-	
Trait Anxiety	0.408 *	−0.595 **	−0.599 **	0.879 **	-

*Note:* ** *p* < 0.01; * *p* < 0.05.

## Data Availability

The original contributions presented in this study are included in the article. Further inquiries can be directed to the corresponding author.
